# Clinical characteristics and treatment for laryngeal cyst in children: a case series study

**DOI:** 10.3389/fsurg.2025.1469405

**Published:** 2025-08-26

**Authors:** Pei Zhou, Lili Zhong, Xiaofang Ding, Mohammd Ashkan Moslehi, Silan Liu, Li Peng, Han Huang

**Affiliations:** ^1^ Children’s Medical Center, Hunan Provincial People’s Hospital, The First Affiliated Hospital of Hunan Normal University, Changsha, China; ^2^ Director of Pediatric Interventional Pulmonology Division, Shiraz University of Medical Sciences, Shiraz, Iran

**Keywords:** laryngeal cyst, children, bronchoscopy, ablation, case series

## Abstract

**Purpose:**

This study aims to present a case series of pediatric laryngeal cysts and summarize their clinical characteristics and treatment outcomes.

**Methods:**

This retrospective case series study included pediatric patients with laryngeal cysts at the author's Hospital, and clinical data were analyzed.

**Results:**

A total of 20 patients were included. Among them, 15 children were diagnosed with supraglottic cysts, while five were categorized into the glottic and subglottic groups. The primary symptoms included stridor (16/20, 80%), shortness of breath (15/20, 75%), coughing after feeding (12/20, 60%), cyanosis (7/20, 35%), and Spo^2^ < 95% (8/20). Bronchoscopy revealed single or multiple transparent or semitransparent cystic masses capable of causing varying degrees of compression of the epiglottis or airway obstruction. Thirteen children with supraglottic laryngeal cysts underwent endoscopic plasma cyst ablation, while five children in the glottic and supraglottic areas underwent bronchoscopic titanium laser ablation. Almost all patients showed significant symptom improvement after treatment. None of the other children (18/20, 90%) experienced a recurrence during the 2 to 36-month follow-up period.

**Conclusion:**

Bronchoscopy should be considered for children experiencing stridor, shortness of breath, coughing after feeding, cyanosis, and Spo^2^ < 95%. Treatment choice between endoscopic plasma cyst ablation and bronchoscopic titanium laser ablation depends on cyst type.

## Introduction

Laryngeal cysts, initially reported by Verneuil in 1,852, are a rare underlying cause of recurrent stridor in infants and young children, with an incidence rate of 1.87 per 100,000 (1). Laryngeal cysts typically manifest with non-specific clinical symptoms such as stridor, dyspnea, and cough. Laryngoscopy serves as the primary initial diagnostic modality for detecting laryngeal cysts (2). However, as most patients are infants and young children with limited communication abilities, they are at risk of being misdiagnosed with conditions like laryngomalacia, laryngitis, or pneumonia (3). Without prompt diagnosis and appropriate treatment, laryngeal cysts can lead to recurrent choking, feeding difficulties, and delayed development in affected children. In severe cases, they can result in significant hypoxemia, respiratory failure, or even fatalities due to cyst compression or airway obstruction (2, 4–6). Therefore, it is crucial for clinicians to accurately recognize the initial symptoms of laryngeal cysts and perform laryngoscopy for the diagnosis of infants and young children.

Laryngeal cysts are a relatively uncommon condition, and reaching a consensus on their surgical management has been challenging. Traditional treatment approaches involve external interventions. Asymptomatic cases may either be monitored or undergo surgical excision. Symptomatic laryngeal cysts are primarily managed through various methods, including external surgical excision, endoscopic plasma cyst ablation, and cyst aspiration (7–9). External approaches offer certain advantages such as excellent exposure of laryngoceles, precise procedures, and low recurrence rates. However, they come with disadvantages, including skin scarring, increased morbidity, longer surgical durations, extended hospital stays, and higher costs (7). A study conducted by Kumar et al. demonstrated that endoscopic radiofrequency ablation effectively and safely removes recurrent saccular cysts, providing improved surgical precision, minimal bleeding, and enhanced healing (10). Cyst fluid aspiration can offer temporary relief for the condition and is often used to alleviate acute upper respiratory tract obstructions. While it is simple and quick, it is prone to relapse and cannot serve as a definitive solution (11).

Currently, there is a need for improvements in the diagnosis and treatment of pediatric laryngeal cysts. However, there is a shortage of published reports on pediatric laryngeal cysts. This study aims to provide a report based on pediatric cases and summarize their clinical characteristics and treatment outcomes.

## Methods

### Study design and patients

This retrospective case series study included pediatric patients diagnosed with laryngeal cysts who were admitted to the author's Hospital for endoscopic therapy between September 2012 and June 2022. The inclusion criteria were as follows: (1) patients aged 0–14 years; (2) laryngeal cysts were confirmed by bronchoscopy and laryngeal imaging examinations; (3) patients who underwent endoscopic therapy. The requirement for individual Informed consent was waived by the First Affiliated Hospital of Hunan Normal University because of the retrospective nature of the study.

### Data collection

Clinical data were retrospectively analyzed, including gender, full-term birth, history of intubation, and symptoms (stridor, cough after feeding, shortness of breath, cyanosis, Spo^2^ < 95%). Additionally, the number and location of laryngeal cysts, imaging results, bronchoscopy examination findings, and treatment methods were collected. Patients were categorized into supraglottic area, glottic area, or subglottic area based on the location of the laryngeal cysts relative to the vocal cords. Children with subglottic cysts involving other areas were grouped into the subglottic area category. Treatment for laryngeal cysts included plasma ablation under self-retaining laryngoscopy, bronchoscopic electrocoagulation through bronchoscopy, bronchoscopic titanium laser ablation.

### Statistical analysis

The data are presented as numbers or percentages, and only descriptive statistics were employed in this study.

## Results

A total of 25 patients diagnosed with laryngeal cysts were initially enrolled. Among them, four patients who exhibited no clinical manifestations and did not receive treatment, and one case with incomplete medical records were subsequently excluded. Ultimately, 20 patients (13 males and 7 females) were included in the study. Five of these patients (25%) had a history of intubation and were born prematurely. Common presenting symptoms included stridor (16/20, 80%), shortness of breath (15/20, 75%), coughing after feeding (12/20, 60%), cyanosis (7/20, 35%), and Spo^2^ < 95% (8/20). Fifteen cases (75%) involved supraglottic laryngeal cysts, one (1/20, 5%) had glottic laryngeal cysts, and four (5/20, 20%) had subglottic laryngeal cysts (Table 1). Eighteen patients (90%) had complications and ten (50%) had laryngomalacia and/or tracheomalacia. Among them, fifteen (75%) had been previously misdiagnosed with conditions such as pneumonia (7/20, 35%), laryngeal cartilage dysplasia (6/20, 30%), laryngitis (1/20, 5%), and bronchopulmonary dysplasia (1/20, 5%), before undergoing bronchoscopy.

**Table 1:** Clinical data of 20 patients with laryngeal cyst.

Patients	Gender	Full-term birth	History of intubation	Number and location of cysts	Treatments
1	F	No	Y	Multiple occurrences in the subglottic region and airway, with the largest located behind the right vocal cord.	1
2	F	No	Y	One, subglottic area	1
3	M	No	Y	Multiple occurrences in the glottic and subglottic region	1
4	M	No	Y	Two, subglottic and tongue root area	1
5	M	No	Y	One, glottic area	1
6	F	Yes	N	One, tongue root	2
7	M	Yes	N	One, epiglottis	3
8	M	Yes	N	One, epiglottis	4–2
9	F	Yes	N	One, epiglottis	2
10	M	Yes	N	One, epiglottis	2
11	M	Yes	N	One, epiglottis	2
12	M	Yes	N	One, epiglottis	4–2
13	M	Yes	N	One, epiglottis	2
14	M	Yes	N	One, tongue root	2
15	M	Yes	N	One, tongue root	2
16	F	Yes	N	One, tongue root	2
17	M	Yes	N	One, tongue root	2
18	M	Yes	N	One, tongue root	3
19	F	Yes	N	One, tongue root	2
20	F	Yes	N	One, tongue root	2

(1): titanium laser therapy under bronchoscopy; (2): plasma cyst ablation under self-retaining laryngoscopy; (3): electrocoagulation under bronchoscope; (4): cyst aspiration procedure.

**Figure 1:**
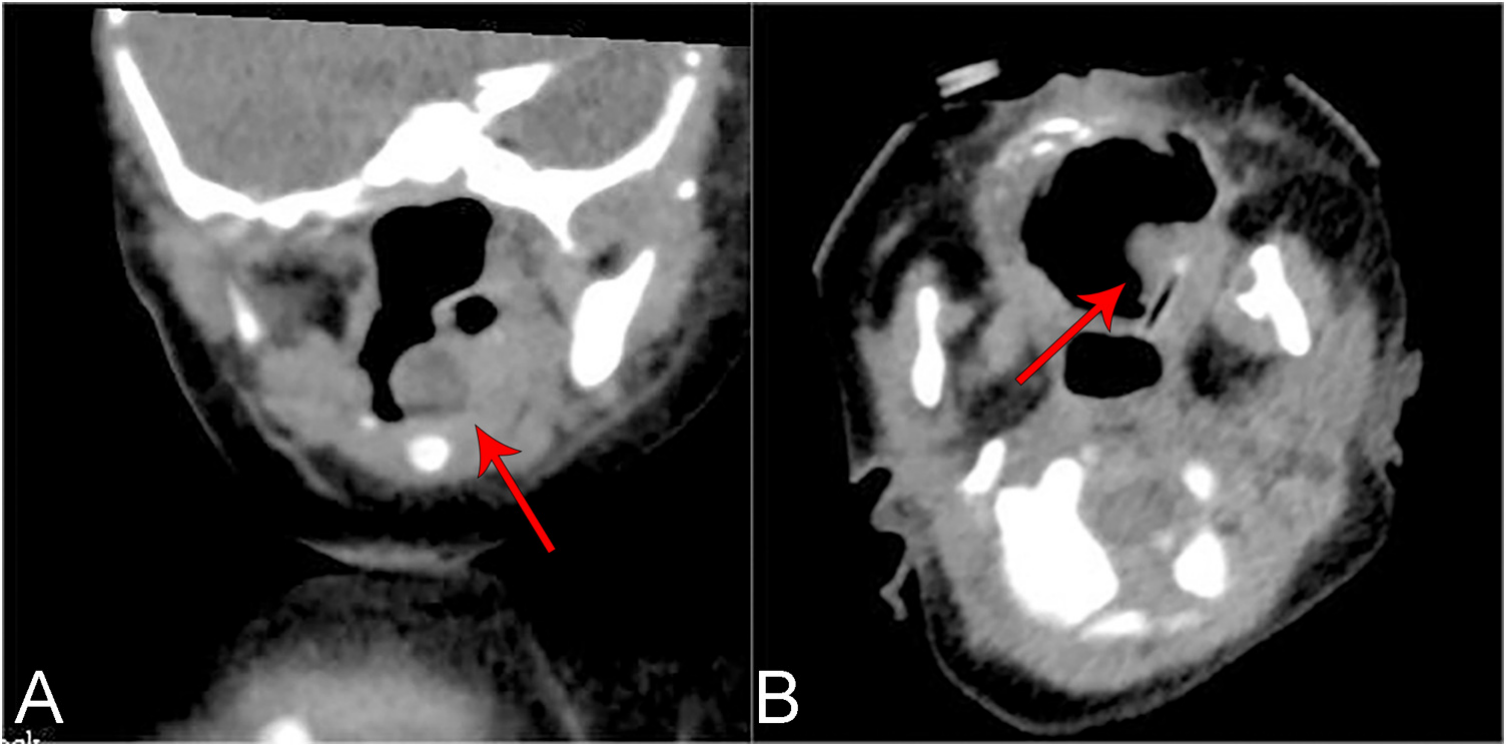
Ct plain scan of the laryngeal cyst of the 19th child in table 2 showed a kind of round slightly low-density shadow with a clear boundary, about 7 × 7 mm in size, and the throat cavity became narrow. **(A)** Coronal plane. **(B)** Cross section.

**Figure 2:**
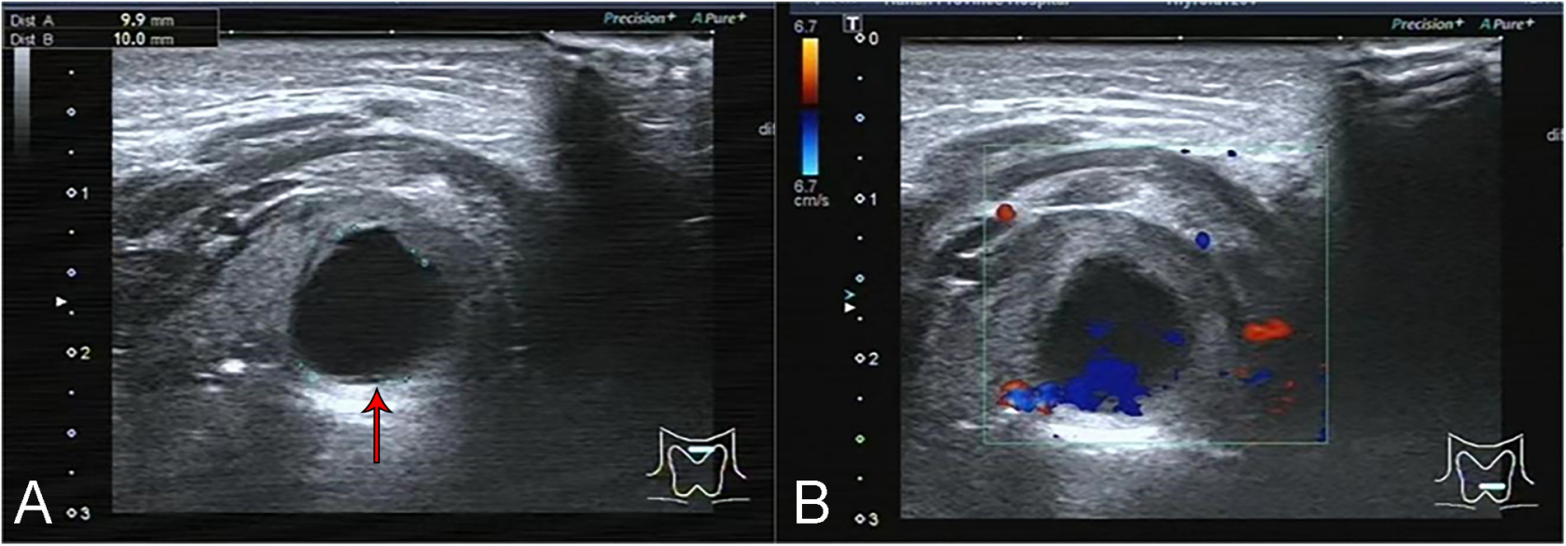
Ultrasound images of the neck of the 19th child in table 2. **(A)** The cyst appeared on ultrasound as a 9.9 × 10.0 mm anechoic zone with clear borders. **(B)** There was no blood flow signal in the cyst.

Seventeen patients (85%) underwent preoperative laryngeal CT scans. The CT scans of the larynx revealed round or quasi-circular low-density lesions with clear boundaries from the surrounding tissue, and no enhancement was observed (Figures 1A,B). Additionally, two patients underwent neck ultrasound examinations, which depicted the lesions as anechoic to hypoechoic areas with regular contours and no blood flow signals (Figures 2A,B). Bronchoscopy was performed on all twenty patients, revealing either single or multiple transparent or translucent cystic masses with smooth surfaces and soft textures, causing varying degrees of compression on the epiglottis or airway obstruction.

Thirteen children diagnosed with supraglottic laryngeal cysts underwent endoscopic plasma cyst ablation. Cases 8 and 12 underwent cyst aspiration procedures before intubation due to severe dyspnea, while cases 7 and 18 received electrocoagulation through bronchoscopy. Additionally, five children with cysts in the glottic and supraglottic areas underwent bronchoscopic titanium laser ablation. In case 9, shortness of breath and a decrease in oxygen saturation to 88% were observed three hours after surgery, necessitating mechanical ventilation, epinephrine administration, and nebulized budesonide. These interventions resulted in the patient's improvement. Fourteen patients received treatment for anti-reflux, either erythromycin (10 mg/kg.d) or omeprazole (0.6–0.8 mg/kg.d), with a median treatment duration of 7 (2–18) days, and this treatment have yielded positive results.

**Figure 3:**
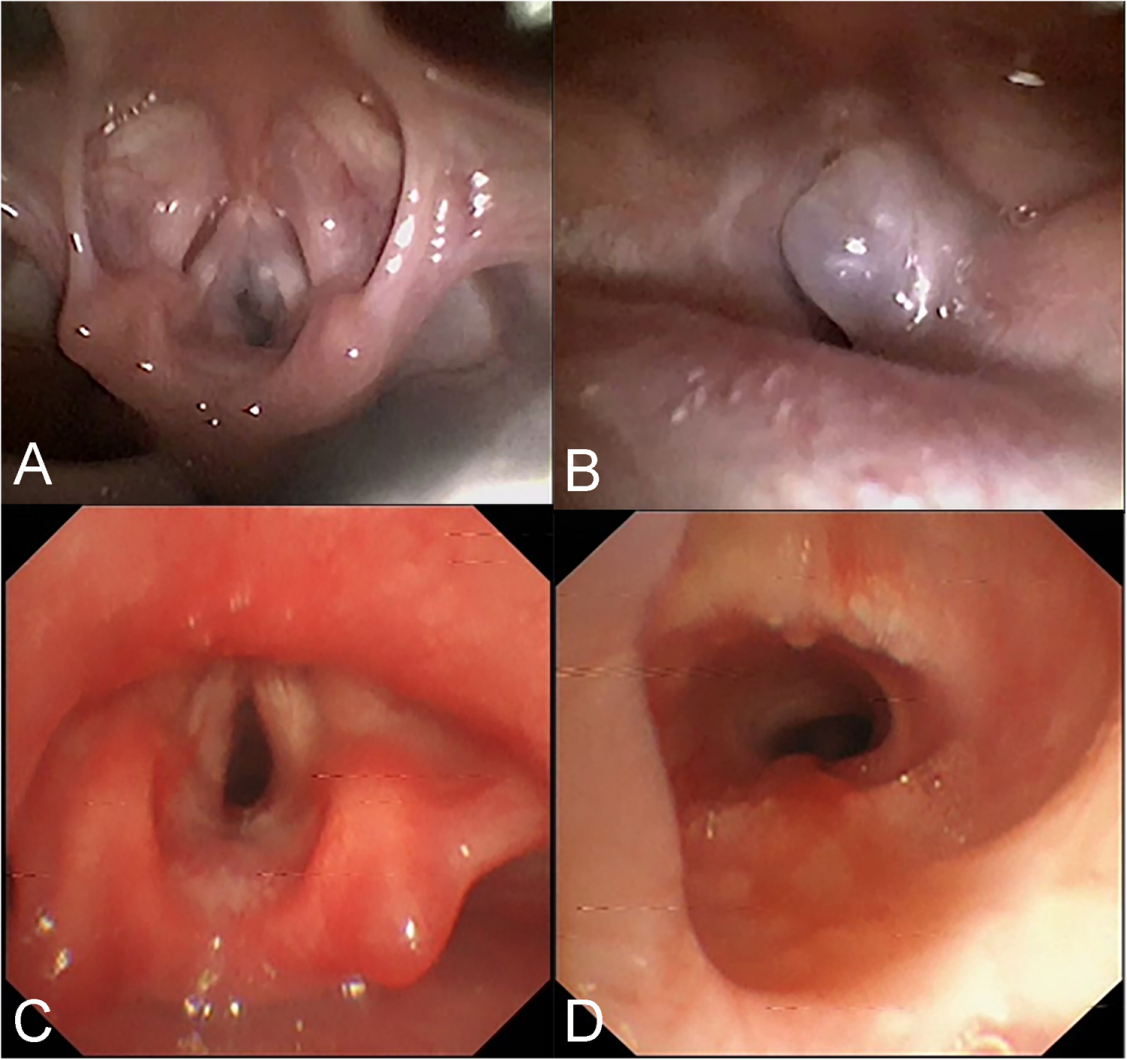
Bronchoscopic images of the 3rd child in table 2 before and after intervention. **(A,B)** A grey transparent cyst can be seen in the right subglottic area, obstructing the airway. **(C,D)** 2.7 months after laser treatment, no residue and stenosis were found in the subglottic area.

During the one-year follow-up for case 2, a new cyst was detected, and two cryotherapy treatments were administered. Subsequently, the mucosa was observed to become smooth and flat at the 18-month follow-up. In case 3, the second-week follow-up examinations revealed a substantial amount of necrotic debris adhering to the affected area, which was then cleared using forceps. However, slight adhesions and scar formation were observed again at the 2-month follow-up. After undergoing laser adhesiolysis and scar release, the mucosa was observed to become smooth at 2.7 months (Figure 3). Telephone follow-ups were conducted over a range of 2 to 36 months, and none of the other children experienced a recurrence.

## Discussion

This study suggests that primary symptoms of laryngeal cysts include stridor, shortness of breath, coughing after feeding, cyanosis, and Spo^2^ < 95%. Children with supraglottic laryngeal cysts underwent endoscopic plasma cyst ablation, while those with cysts in the glottic and supraglottic areas underwent bronchoscopic titanium laser ablation. Almost all patients exhibited significant symptom improvement after treatment. These findings hold considerable clinical significance and can enhance the understanding of this disease among pediatric physicians.

Laryngeal cysts represent a rare cause of stridor and shortness of breath in children. Advances in medical technology, including the development of laryngoscopy and bronchoscopy, have contributed to an increased detection rate of laryngeal cysts (3, 5). Nevertheless, many cases continue to receive misdiagnoses such as laryngomalacia, laryngitis, or pneumonia (3). When left untreated, laryngeal cysts can result in fatal suffocation. A published report indicates a mortality rate of up to 40% for this condition (12). Among the 20 cases in this present study, 15 were initially misdiagnosed. Endoscopy was employed to confirm the diagnosis and locate laryngeal cysts in all 20 patients. Subsequently, a series of interventions, including endoscopic plasma cyst ablation, bronchoscopic titanium laser ablation, mechanical ventilation, epinephrine, nebulized budesonide, and other strategies, led to a complete cure for the majority of patients, with no recurrence observed during the follow-up period.

The findings suggest that laryngeal injury or infection following intubation may constitute a risk factor for the development of laryngeal cysts. As a prior study, laryngeal cysts can be categorized into two groups: congenital and acquired (13). Congenital laryngeal cysts are believed to arise from the failure to maintain the patency of the laryngeal saccules' opening, leading to the accumulation of mucus and subsequent enlargement (14). Acquired laryngeal cysts are primarily associated with laryngeal injury or infection following intubation, where the mechanism might involve the blockage of the laryngeal saccule's opening due to intubation or infection, leading to cyst development (15, 16). Studies have reported the frequent occurrence of subglottic cysts in premature infants with a history of intubation (5). In this study, 15 children presented with laryngeal cysts in the supraglottic area, all of whom were full-term infants without prior intubation. Additionally, five patients had laryngeal cysts in the glottic and subglottic area, all of whom were preterm infants with a history of intubation. Nevertheless, some studies have suggested that premature birth and intubation are not obligatory factors for the development of subglottic cysts (17). Hence, a more comprehensive analysis of medical records is necessary to further explore the etiology and mechanism of laryngeal cysts.

The clinical manifestations of patients with laryngeal cysts are closely linked to the size and location of the cyst (18). Laryngeal cyst symptoms are influenced by various factors. While a small laryngeal cyst may remain asymptomatic, its size should not be underestimated, as it can exert significant pressure on surrounding tissues (19). When large, it can obstruct the laryngeal inlet, resulting in acute airway obstruction, which can be life-threatening. Furthermore, the location of the laryngeal cyst is another crucial determinant. Previous studies have indicated that laryngeal cysts most commonly occur in the epiglottis (epiglottic fovea, aryepiglottic folds, epiglottis, arytenoid cartilage, and laryngeal pouch), but they are less frequent in the glottis and subglottic areas (1). Zawadzka-Glos et al. reported a higher risk of laryngeal cyst occurrence in the subglottic area among intubated premature infants (19), aligning with the findings of this study. Laryngeal lesions directly impact the function of surrounding tissues, leading to clinical manifestations such as stridor, shortness of breath, and choking during the ingestion of liquids (20).

Laryngeal cysts often present with non-specific symptoms, which can make it necessary to distinguish them from conditions like laryngomalacia or laryngitis (18). Laryngomalacia is the leading cause of recurrent stridor in infants and young children (21). On the other hand, laryngitis is frequently a consequence of viral or bacterial infections, characterized by an acute onset, and its clinical features mainly include hoarseness, a barking cough, and inspiratory stridor and dyspnea, which can be rapidly relieved with anti-infection therapy and glucocorticoid nebulization (22). In our study, common patient presentations included stridor (16/20, 80%), shortness of breath (15/20, 75%), coughing after feeding (12/20, 60%), cyanosis (7/20, 35%), and Spo^2^ < 95% (8/20). These symptoms can significantly contribute to misdiagnosis. If these symptoms manifest shortly after birth or worsen with activities, crying, or lying on the back, and show little improvement after symptomatic treatment, early bronchoscopy or electronic laryngoscopy is strongly recommended (5, 17). For children suspected of having supraglottic cysts, some studies recommend flexible laryngoscopy due to its advantages in terms of flexibility, light transmission, a wide field of vision, and minimal stimulation (23), despite its limitation in identifying subglottic and lower cysts. Among the 20 cases included in this study, 18 had concurrent pneumonia. If necessary, alveolar lavage therapy could be performed during bronchoscopy. Therefore, for patients with an uncertain localization of the cyst or concurrent pulmonary infection, a bronchoscopy test is recommended as the primary diagnostic approach.

Endoscopy alone cannot accurately measure the size of the cyst or determine its nature and anatomical relationship with surrounding tissues. Therefore, before interventional treatment, it is necessary to complete comprehensive neck imaging examinations. Among these, Laryngeal CT (plain scanning and contrast-enhanced CT) is the most commonly used modality, aiding in differentiating laryngeal cysts from conditions with pathological changes that have a rich blood supply, such as hemangiomas (24). Laryngeal cysts typically appear as low-density lesions without enhancement on contrast-enhanced CT, while hemangiomas exhibit significant enhancement. They often manifest as low signals on T1-weighted images and high signals on T2-weighted images, with no enhancement observed when using gadolinium contrast agent in Magnetic Resonance Imaging (MRI). Previous studies have shown that MRI is valuable in diagnosing the extent and type of cystic lesions and can distinguish between fluid-filled and air-filled laryngeal cysts (23, 25). However, MRI examinations often require the use of sedatives, which can be challenging for infants and young children and carry the risk of upper airway obstruction. Studies have demonstrated that laryngeal ultrasound, particularly high-resolution ultrasound, is as effective as laryngeal CT and MRI in diagnosing and differentiating laryngeal diseases (4, 26). Additionally, ultrasound offers the advantages of convenience, absence of radiation, and no need for sedation. Cysts typically appear as anechoic to hypoechoic areas on ultrasound with well-defined, regular, and clear contours, and no blood flow signals are detected. However, it's worth noting that the quality of the ultrasound examination can be influenced by the technician's experience and machine performance, potentially leading to an increased rate of missed diagnoses. In summary, for children suspected of having laryngeal cysts, a combination of endoscopy and imaging examinations such as laryngeal CT, MRI, and ultrasound should be performed to facilitate diagnosis. The treatment methods for laryngeal cysts mainly include conservative management, cyst aspiration, endoscopic low-temperature plasma radiofrequency ablation (LTP-RFA), EC therapy, laser therapy, and open surgical approaches. For children with small cysts that do not cause obstructive symptoms, conservative treatment with regular follow-up can be a viable option, as these cysts may spontaneously resolve or decrease in size over time (17). Cyst aspiration is a straightforward procedure that rapidly relieves airway obstruction symptoms and is recognized as an effective emergency procedure (2). In this study, cases 8 and 12 presented with pronounced dyspnea and cyanosis, which promptly improved following aspiration. However, a notable drawback is the relatively high recurrence rate compared to other treatment modalities (6). Hence, interventional surgery remains necessary to effectively disrupt the cystic wall. Endoscopic treatments are suitable for cases with thin cyst walls located within the larynx, and among these methods, low-temperature plasma radiofrequency ablation (LTP-RFA) has proven effective in treating lesions in the supraglottic region (6). The basic mechanism involves high-reactive free radicals in low-temperature plasma leading to tissue disintegration, generating carbohydrates and oxygen ions, and resulting in tissue coagulative necrosis (27). Among the 15 children with supraglottic laryngeal cysts in this study, 13 received low-temperature plasma cyst ablation using a self-retaining laryngoscope, while two underwent bronchoscope-guided EC therapy. This study demonstrated that compared to the latter method, LTP-RFA offers advantages such as better visualization, shorter surgical time, smoother incisions, and avoidance of tissue swelling and carbonization damage caused by the instantaneous high temperature of electrocoagulation. Titanium laser ablation treatment offers advantages such as high accuracy, minimal damage to surrounding tissues, favorable hemostatic properties, and low recurrence rates (28). This method is commonly employed in infants and young children with cysts located in the narrow glottis and subglottic region. In the case of patient 3, multiple cysts were located in the glottis and subglottic areas. Subsequent fiberoptic bronchoscopy conducted at 1 and 2 weeks post-surgery revealed a considerable amount of necrotic debris, with adhesions and scar formation observed after its removal with forceps. Therefore, for pediatric patients with multiple laryngeal cysts, it may be advisable to minimize damage to normal tissues during the procedure by adopting moderate laser ablation or sequential ablation and forceps to reduce postoperative wound congestion, edema, and necrosis. Follow-up visits should be appropriately increased in frequency and duration. Postoperative combined therapy of anti-inflammation, anti-infection, anti-reflux, and nebulization may be beneficial in preventing laryngeal edema, airway spasm, and scar formation, among other complications. The postoperative combination therapy of anti-inflammation, anti-infection, anti-reflux, and nebulization may play a beneficial role in preventing complications such as laryngeal edema, airway spasms, and scar formation. In this study, anti-reflux treatment may have positively influenced wound site recovery, potentially reducing the risk of scar formation and recurrence. Laryngeal cysts have been reported to co-occur with laryngomalacia (29, 30). While anti-reflux medications have been reported to treat laryngomalacia, the evidence supporting their use as a therapy for laryngomalacia is of low quality due to potential biases in patient selection and the absence of comparator groups (31). In this study, omeprazole was administered to 14 patients as an anti-reflux treatment, and this intervention have yielded positive results, supporting the efficacy of anti-reflux therapy in patients with laryngeal cysts.

This study presents several limitations. Firstly, it is a retrospective case series with a limited sample size, primarily due to the rarity of laryngeal cysts in children. Consequently, the possibility of chance leading to spurious results cannot be entirely excluded. Secondly, the cases were exclusively obtained from a single hospital; therefore, generalizing the results may require additional research.

In conclusion, bronchoscopy should be considered for children experiencing symptoms such as stridor, shortness of breath, coughing after feeding, cyanosis, and Spo^2^ < 95%. The choice between endoscopic plasma cyst ablation and bronchoscopic titanium laser ablation should be based on the specific type of laryngeal cyst.

## Data Availability

The original contributions presented in the study are included in the article/Supplementary Material, further inquiries can be directed to the corresponding author.
